# Bacteriophage ZCSE2 is a Potent Antimicrobial against *Salmonella enterica* Serovars: Ultrastructure, Genomics and Efficacy

**DOI:** 10.3390/v12040424

**Published:** 2020-04-09

**Authors:** Ahmed Mohamed, Omar Taha, Hesham M. El-Sherif, Phillippa L. Connerton, Steven P.T. Hooton, Nabil D. Bassim, Ian F. Connerton, Ayman El-Shibiny

**Affiliations:** 1 Center for Microbiology and Phage Therapy, Biomedical Sciences, Zewail City of Science and Technology, Giza 12578, Egypt; ahmed.mostafa.taha@gmail.com (A.M.); p-okhaled@zewailcity.edu.eg (O.T.); 2 Mechanical Design and Production Engineering Department, Faculty of Engineering, Cairo University, Giza 12613, Egypt; elsherih@mcmaster.ca; 3 Materials Science and Engineering Department, McMaster University, 1280 Main Street West, Hamilton, ON L8S 4L8, Canada; bassimn@mcmaster.ca; 4 Division of Microbiology, Brewing and Biotechnology, School of Biosciences, University of Nottingham, Loughborough LE12 5RD, UK; pippa.connerton@nottingham.ac.uk (P.L.C.); sbzsph@exmail.nottingham.ac.uk (S.P.T.H.); 5 Faculty of Environmental Agricultural Sciences, Arish University, Arish 45615, Egypt

**Keywords:** bacteriophage, *Salmonella*, phage biocontrol, atomic force microscopy

## Abstract

Developing novel antimicrobials capable of controlling multidrug-resistant bacterial pathogens is essential to restrict the use of antibiotics. Bacteriophages (phages) constitute a major resource that can be harnessed as an alternative to traditional antimicrobial therapies. Phage ZCSE2 was isolated among several others from raw sewage but was distinguished by broad-spectrum activity against *Salmonella* serovars considered pathogenic to humans and animals. Lytic profiles of ZCSE2 against a panel of *Salmonella* were determined together with low temperature activity and pH stability. The morphological features of the phage and host infection processes were characterized using a combination of transmission electron and atomic force microscopies. Whole genome sequencing of ZCSE2 produced a complete DNA sequence of 53,965 bp. No known virulence genes were identified in the sequence data, making ZCSE2 a good candidate for phage-mediated biological control purposes. ZCSE2 was further tested against *S.* Enteritidis in liquid culture and was observed to reduce the target bacterium to below the limits of detection from initial concentrations of 10^7^–10^8^ Colony Forming Units (CFU)/mL. With a broad host-range against pathogenic *Salmonella* serovars, phage ZCSE2 constitutes a potential tool against a major cause of human and animal disease.

## 1. Introduction

The global disease burden caused by *Salmonella* spp. represents a significant proportion of microbial infections acquired through the consumption of contaminated food and water. In the developed world, *Salmonella* infection usually presents as a self-limiting gastrointestinal infection, although antibiotic treatments may be required. Non-typhoidal *Salmonella* (NTS) can be attributed to 93 million cases of gastrointestinal disease annually, of which 155,000 infections result in death [1]. Furthermore, invasive non-typhoidal *Salmonella* (iNTS) is associated with enhanced pathogenicity towards specific demographics in the developing world. Notably, the young, elderly or immunocompromised are at risk from iNTS infections, with ≈3.4 million reported cases on an annual basis [2]. Approximately 388,000 deaths arise from iNTS infection in sub-Saharan Africa alone, and globally the reported mortality is greater than 681,000 per annum [3,4]. Most instances of NTS/iNTS disease are associated with the globally distributed zoonotic serovars *Salmonella* Typhimurium and *Salmonella* Enteritidis [5,6]. Antimicrobial resistance (AMR) is well-documented in *Salmonella* spp., with many isolates being identified as being resistant to multiple antibiotic classes [7].

In terms of global food security, foodborne disease arising from consumption of *Salmonella*-contaminated products remains a serious public health issue. Intensive farming practices such as those employed by the poultry production industry provide the perfect environment for the development and spread of AMR. Investigating alternative antimicrobial agents for the control of *Salmonella* and other pathogenic bacteria in food production environments is therefore paramount. One promising strategy is the use of bacteriophages (phages). As a naturally occurring biological control agent, phages represent a valuable tool for the targeted removal of bacteria from the food chain and other environments. Phages can be highly specific in terms of the target bacterial host and are ubiquitous in the environment. Phages have the capacity to self-replicate at an infection focal point at the expense of the host bacterial population. Such properties make phages a viable option for the decontamination of *Salmonella* in food production environments, and with the potential to extend therapeutic applications into clinical settings [8,9,10,11,12]. Many studies have shown that phage biocontrol of *Salmonella* can be achieved, and with products such as SalmoFresh (Intralytix Inc.) and Salmonelex/PhageGuard S (Micreos, BV) being marketed as food processing agents, it is evident that such approaches are efficacious.

Ensuring constituents of phage treatments conform to safety requirements is far from trivial [13]. Undesirable traits including the ability of temperate phages to integrate into bacterial chromosomes or potential transduction of genes during infection events can be avoided through careful analysis of candidate phages in the laboratory. Obtaining complete genome sequences is also a prerequisite for phages being used in laboratory studies and beyond. Environmental stability of phages is also an important factor to consider. Some phages may only retain activity within a given pH range, which may limit their use in certain applications; however, for storage purposes, retaining phage infectivity following long-term storage in neutral buffers is essential.

Complementary to the standard phage experiments that are used to monitor lifecycles, new approaches are becoming available that allow visualization of phage interactions with their microbial hosts. Advances in techniques such as atomic force microscopy (AFM) have allowed the infection process and lifecycle characteristics of phage AP22 during lytic growth on *Acinetobacter baumannii* to be visualized [14]. AFM was used to determine phage morphology, estimate the phage latent period, and further allowed detection of morphological changes at different stages of the lytic cycle by monitoring host cell adsorption. Various AFM scanning modes can be applied to study phages [15]. The non-contact mode of AFM is preferred in imaging phages because it provides no physical interaction between the sample and the scanning tip. However, the contact mode can be applied to study the mechanics of phages and used to monitor the infection process [16]. AFM peak-force mode has been used to manipulate phages using a chemically functionalized AFM tip [17].

The current paper aimed to characterize a novel virulent phage ZCSE2 that appears to be a representative of a phage class that has antimicrobial potential against multiple *Salmonella* serovars but for which little genetic or structural information is available. We used TEM and AFM imaging to examine the infection characteristics of the phage to establish the suitability for biocontrol applications.

## 2. Materials and Methods

### 2.1. Bacterial Strains and Growth Media

Studies were conducted using multidrug-resistant *Salmonella* Enteritidis WT (Platten) obtained from The University of Nottingham (United Kingdom), where it was used in previous studies [8]. Stocks were maintained in 20% (*v*/*v*) glycerol at −80 °C until needed. Bacterial strains were grown on Tryptic Soy agar (TSA; Oxoid, England) overnight at 37 °C and phage infections were carried out in Tryptic Soya broth (TSB; Oxoid, England) at 37 °C with shaking.

### 2.2. Antibiotic Sensitivity Test

The resistance profile of *S.* Enteritidis WT (Platten) was determined using a panel of antibiotics (Oxoid, England) representative of those administered to humans and animals (Table 1). Antimicrobial sensitivity testing was performed using the disk diffusion methods in accordance with the National Committee for Clinical Standards guidelines [18].

### 2.3. Bacteriophage Isolation, Amplification, and Purification

Phages were isolated from environmental samples taken from waterways in El-Marg, Cairo, Egypt. *S.* Enteritidis WT (Platten) was used as a host bacterial strain for phage isolation. Phage plaques were purified by repeated single plaque isolation using sterile micropipette tips for a minimum of three rounds to obtain pure phage stocks [19]. All isolated phages were amplified to generate high-titer stocks as follows: 100 mL of host bacterium (10^7^ CFU/mL) was infected with phages at a multiplicity of infection of 0.1 and allowed to lyse bacteria overnight in TSB (Oxoid, United Kingdom) at a temperature of 37 °C on an orbital shaker at 120 rpm [20]. Lysates were centrifuged at 6400× *g* for 15 min at 4 °C to remove remaining bacterial cells. The supernatant containing phages was then centrifuged for at 15,300× *g* for 1 h at 4 °C. Phage pellets were resuspended in SM buffer (100 mM MgSO_4_/7H_2_O; 10 mM NaCl; 50 mM TrisHCl; pH 7.5) prior to filtration with 0.22 µm syringe filters (Chromtech, Taiwan). Phage titers were determined using standard double-agar overlay plaque assays [21]. Briefly, a single bacterial colony was picked using a sterile loop and used to inoculate TSB prior to incubation at 37 °C with shaking for several hours. From this culture, 100 µL was added to 3 mL of 1% Bacto top agar in Ok TSB (≈55 °C) prior to pouring onto TSA plates. After solidification, 10 µl aliquots of 10-fold serial-diluted ZCSE2 were spotted in triplicate onto the bacterial lawn. Phage titers were calculated following overnight incubation at 37 °C.

### 2.4. Lytic Profile of Isolated Phages

Isolated phages were tested against an in-house panel of 25 *Salmonella* serovars. Criteria for selection involved identifying phages displaying a broad range of lysis against the *Salmonella* panel, alongside the ability to produce clear plaques on the host bacterium. Using double agar overlay plaque assays as described above, the lytic profile of isolated phages was determined against a panel of *Salmonella* strains shown in Table 2. Initial phage titers applied to lawns were not less than 10^9^ plaque forming units (PFU)/mL in 10 µL per spot equating to a routine test dilution of 10^7^ PFU. The lytic activity of phages was determined on the basis of zones of clear lysis.

### 2.5. Efficiency of Plating

Over eight decimal dilutions, bacteriophage ZCSE2 was tested in triplicate against all the susceptible bacterial isolated that were lysed in spot assays as previously described [22]. Using the same conditions as spot test, 200 microliters of bacterial isolates in Log-phase were added to top agar at a temperature of 55 °C. The top agar containing bacteria was added to Petri dishes of TSA and allowed to set and dry, before 10 μL of serially diluted phage were dispensed onto the surface and the plates were incubated overnight at 37 °C. Efficiency of plating (EOP) was then calculated as the average PFU on target bacteria/average PFU on host bacteria.

### 2.6. Phage Genome Size Determination Using (PFGE)

DNA was prepared from phage ZCSE2 (10^10^ PFU/mL) for genome size determination by PFGE [23]. Briefly, ZCSE2 was suspended in 1.2% agarose plugs prior to digestion with lysis buffer (0.2% *w*/*v* SDS; 1% *w*/*v N*-lauryl sarcosine; 100 mM EDTA; 1 mg/mL proteinase K), overnight at 55 °C. Following washing, 2 mm slices of agarose-containing DNA were inserted into the wells of a 1% *w*/*v* agarose gel. The gel was run by using a Bio-Rad CHEF DRII system, in 0.5 × Tris-borate-EDTA, for 18 h at 6 V/cm with a switch time of 30 to 60 s. The size of the genome was determined by comparison to standard concatenated lambda DNA markers (Sigma Aldrich, Gillingham, United Kingdom).

### 2.7. In Vitro ZCSE2 Lytic Activity

The survival of a growing culture of *S.* Enteritidis WT (Platten) in the presence of ZCSE2 phage at multiplicities of infection (MOI) of 0.1, 1, and 10 PFU/CFU was estimated in comparison to uninfected bacterial control (phage-free samples) at 37 °C [24]. Phage infective centers (IC) and plaque forming units (PFU) were also estimated at different time intervals post addition (0, 5, 10, 20, 30, 40, 60, 90, 120, and 180 min). IC is the amount of free phage particles already released from the bacterial cells, without adding chloroform, whereas PFU describes the amount of nascent phage both inside and outside the bacterial cell. Briefly, two flasks were filled with either bacterial culture at a given concentration (control) or with bacterial culture at the same concentration and bacteriophage matching the desired MOI (Test). At every time interval, titer of bacterial control (B), bacterial survival (BS) IC and PFU were simultaneously estimated. Bacterial titer were determined using the Miles and Misra method [25], whereas phage titer was estimated using double-agar overlay plaque assays by adding chloroform to the aliquot to be estimated in case of PFU determination, or not adding chloroform to calculate the IC.

### 2.8. Bacteriophage Insensitive Mutant Frequency

The frequency of insensitive mutants was evaluated as follows. Phages were used to infect bacterial cultures at a multiplicity of infection (MOI) of 100 and incubated for 10 min at 37 °C. The infected cultures were serially diluted, and the survivor frequency estimated as the CFU of survival colonies per CFU of initial colonies after overnight incubation at 37 °C [26]. Experiments were performed in triplicate and standard deviations were calculated. The surviving colonies were picked, and from these the bacteriophage-insensitive mutants (BIM) were established by plaque formation on bacterial lawns.

### 2.9. ZCSE2 pH Stability

The viability of ZCSE2 at different pH values (2–9) was determined by enumerating the phage titer as previously described after 1 h incubation of a 10 log_10_ PFU/mL suspension in a range of SM buffer. The range of pH of SM buffer was adjusted by using NaOH or HCl.

### 2.10. ZCSE2 Activity at 4 °C

The antimicrobial effect of ZCSE2 was investigated at low temperature by incubating the phage with *Salmonella* Enteritidis WT (Platten) at 4 °C. The bacterial cells were allowed to grow in TSB to ≈10^7^ CFU/mL and then acclimated at 4 °C for 10 min before the addition of ZCSE2 at MOI 10. Following incubation at 4 °C, samples were collected at 0, 2, 4, and 24 h to enumerate the number of phages (PFU/mL) and bacterial survivors (CFU/mL), as described above.

### 2.11. ZCSE2 Genome Sequencing

A high titer stock of ZCSE2 (≈10^10^ PFU/mL) was prepared as described above. To obtain ZCSE2 genomic DNA, phages were initially treated with proteinase K (100 μg/mL in 10 mM EDTA (pH 8)). DNA was extracted from the preparation using DNA Wizard Kit (Promega, United Kingdom) according to the manufacturer’s instructions. Library preparation of ZCSE2 genomic DNA followed the Illumina Nextera tagmentation protocol (Illumina, Cambridge, United Kingdom) and the library sequenced using the Illumina v3 sequence cassette for 600 cycles on the MiSeq platform. The data were composed of 3.1 million paired-end sequence reads with lengths of 250 bp. De novo assembly of sequence reads was performed using CLC Genomics Workbench version 11.0.1 (Qiagen, Aarhus, Denmark). Assembled reads yielded a complete double-stranded DNA ZCSE2 genome of 53,965 bp (coverage around 7000-fold). Gene predictions were made using PHASTER [27] and HHpred [28] to identify putative open reading frames (ORFs), followed by manual curation and polishing with Artemis and BLAST (non-reductive NCBI databases). The sequence is available under the GenBank accession number MK673511. Bacteriophage genome sequence alignments, average nucleotide identities, and phylogenetic relationships were calculated using CLC Genomics Workbench 20.0.3.

### 2.12. Morphology Investigation by Transmission Electron Microscopy (TEM)

#### 2.12.1. TEM Device Specification and Examination Conditions

Preliminary sample screening was performed using a Philips CM12 microscope at medium magnifications at 120 KeV accelerating voltage using a LaB6 electron gun with parallel illumination and minimized current. The images were collected using an ORIUS SC600 CCD camera with 2.7k × 2.7k pixel size.

After screening, candidate ZCSE2 bacteriophage particles were imaged using an FEI Titan 80–300 Cubed transmission electron microscope (located in the Canadian Centre for Electron Microscopy, Hamilton, Canada) equipped with a monochromator and double hexapole-based spherical aberration correctors in both the probe and the image forming lenses. The Titan microscope operated at an accelerating voltage of 80 keV. Images were acquired in scanning TEM mode at 19.1 mrad conversion angle and 91 cm camera length using both bright field (BF) and high annular angle dark field (HAADF) detectors with 2048 × 2048 pixel size. The imaging conditions were selected to reduce possible electron beam damage of the samples and the electron probe dose lowered to be less than 70 e^−^/Å^2^S and 4 µS dwell time. Several TEM images at low and high magnification were taken from the sample at different locations of the TEM copper grid to locate the phages and observe the most repeatable morphology. In addition to the scanning transmission electron microscopy (STEM) mode, the Titan microscope was also used in TEM mode using parallel illumination in screening and navigation using a US1000FTXP CCD camera.

#### 2.12.2. TEM Sample Preparation

The ZCSE2 was centrifuged at 12,000 rpm for 30 min and resuspended in 100 µL SM buffer. Uranyl acetate, 10 µL of 1% *w*/*v* solution, was used as a negative stain on a TEM carbon lacey grid at room temperature. Residual mixture was removed and, finally, the sample was exposed to light from a warm lamp at 30 cm distance for 15 min at room temperature.

#### 2.12.3. TEM Data Analysis

Several TEM images at high magnification were taken for phages at different locations from three TEM copper grids. To calculate statistical values of the phage main dimensions, ImageJ program (open source) was used to measure the phage head diameter (D), phage tail diameter (d), and the total phage length (L) to calculate the average and standard deviation for these dimensions.

### 2.13. AFM Sample Preparation

For AFM analyses, ZCSE2 with titer of 10^9^ PFU/mL was used. Overnight culture of *S.* Enteritidis WT (Platten) was diluted in fresh liquid culture and incubated for 90 min at 37 °C. The culture was then subdivided into two parts: uninfected control and infected (MOI 50). At different time intervals varying from 1 to 120 min, aliquots of samples were taken and investigated with AFM. Samples of either *S.* Enteritidis cells or mixture with ZCSE2 phage were diluted 10-fold by adding 9 mL of milli-Q water to 1 mL of sample. A drop of 5 µL was dispensed on a highly oriented pyrolytic graphite (HOPG) surface and allowed to dry in a laminar flow hood for 10 min. Residual liquid was removed. The process was repeated 3–5 times before AFM examination.

#### 2.13.1. AFM Device Specification

An AFM neaSNOM microscope (nea-Spec GmbH, Munich, Germany) with 0.2 nm resolution was used to perform both surface topology and phase imaging of bacteria and phages. AFM probes from NANO-WORLD of 45 × 4.6 µm cross section and 160 µm length with 285 KHz average resonance frequency were used. The probes possess a thick coating layer of a 23 nm of platinum-iridium on both sides of the cantilever. The radius of the probe tip is assumed to be less than 10 nm by the manufacturer. However, the radius may change due to particle or liquid adhesion to the tip during the test. In order to optimize the image resolution, several images were taken from the region of interest at different scan speeds and scan sizes. All AFM images were maintained with pixel times more than 5 ms/pixel to acquire strong signal.

#### 2.13.2. Post-Processing of AFM Images

Raw data of topology and phase imaging were processed by open source “Gwyddion” software Version 2.55 (http://gwyddion.net/download.php) [29]. The software was used to level the data by mean plane subtraction and to reduce minor scan defects and scan noise without affecting the physical dimensions of the images. These were used to plot cross sections and height distribution of AFM topology images.

### 2.14. Statistical Analysis

Test and control datasets were compared using Student’s *t*-test. A *p*-value of 0.05 or less was considered statistically significant in all cases. Analytical statistics were calculated using ANOVA with GraphPad PRISM software.

## 3. Results

### 3.1. Bacterial Sensitivity to Antibiotics

To determine the antibiotic resistance of the bacteriophage host *Salmonella* Enteritidis WT (Platten), antibiotic sensitivity tests were performed using the disc diffusion method. *Salmonella* Enteritidis WT (Platten) was resistant to 10 different antibiotics, including cefotaxime, clarithromycin, and vancomycin, whereas it is intermediately resistant to the three antibiotics ciprofloxacin, azithromycin, and cefoxitin. The strain also showed sensitivity to another set of antibiotics, as noted in (Table 1). This demonstrated the high antibiotic resistance profile of the *Salmonella* Enteritidis WT (Platten) strain.

### 3.2. Lytic Profile and EOP of Isolated Bacteriophages

Bacteriophages were isolated from sewage water using multidrug-resistant *Salmonella* Enteritidis WT (Platten) as host. Phage isolates were purified via successive rounds of plaque purification. Following this, testing was performed on different strains of *S. enterica* to select potential lytic phages. Phage ZCSE2 showed the highest capability of lysis against many strains compared to other phages (Table 2). ZCSE2 phage was capable of producing lysis zones (≥20 plaques) at the routine test dilution of 10^7^ PFU on 24 out of 25 *Salmonella* strains representing 16 serotypes (Table 2). ZCSE2 demonstrated a potent antibacterial action against several multidrug-resistant (MDR) virulent *Salmonella* such as *S.* Typhimurium DT104 and *S.* Typhimurium U288 [8,30]. Notably, phages ZCSE4 and ZCSE5 exhibited restricted host ranges and could not be easily propagated on the isolation host. These data indicated that ZCSE2 had broad-spectrum anti-*Salmonella* activity and was selected for further study. The ability of ZCSE2 to lyse multiple *Salmonella* serovars was further assessed by examining the efficiency of plating (EOP). The selected phage exhibited EOPs ≥ 0.5 for 19 of 22 *Salmonella* isolates examined (Table S1).

### 3.3. Antibacterial Efficacy of ZCSE2 In Vitro

**Figure 1:**
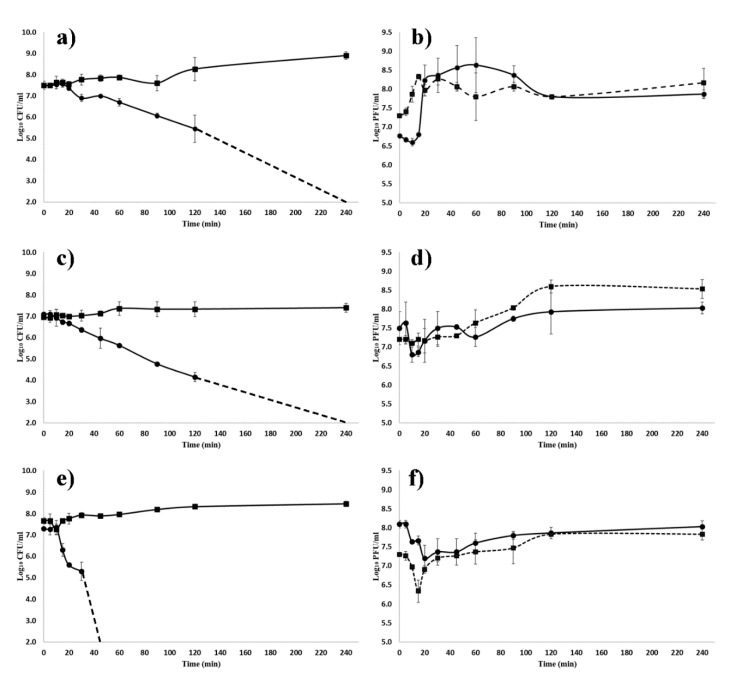
The kinetics of ZCSE2 infection of *Salmonella* Enteritidis WT (Platten) at 37 °C. (**a**,**c**,**e**) Bacterial counts for control- (■) and phage-infected (●) cultures, where the dashed lines indicate when the viable count fell below the limit of detection of 2 log_10_ CFU/mL for *Salmonella*. (**b**,**d**,**f**) Phage titers for infective centers (log_10_ plaque forming units (PFU)/mL) (●; solid line) and nascent phage (log_10_ PFU/mL) (■; dashed line). Panels (**a**,**b**) show a starting multiplicities of infection (MOI) 0.1; panels (**c**,**d**) starting MOI 1; panels (**e**,**f**) starting MOI 10.

To quantify the antibacterial lytic activity of ZCSE2, growing cultures of *S.* Enteritidis WT (Platten) were infected with ZCSE2 at different MOIs of 0.1, 1, and 10 over a period of 4 h. The infection and lysis characteristics, including time to lysis and burst size for ZCSE2, were quantified (Figure 1a–f). The period of infection to the onset of lysis was estimated to be in a range of 15 to 30 min for each MOI. The estimate of burst size for MOI 0.1 was 155 PFU/CFU. *Salmonella* Enteritidis WT (Platten) was lysed by ZCSE2 at each MOI tested, with a MOI of 10 reducing viable bacteria from 7.5 log_10_ CFU/mL to below the limit of detection (2 log_10_ CFU/mL) at 37 °C after 45 min (Figure 1e). Log_10_ reductions in bacterial counts compared to control were recorded after 60 min for MOIs 1 and 0.1 PFU/CFU, and fell below the detection limit after 120 min. Notably, the bacterial populations did not recover over the 4 h course of the experiment. The data suggest that ZCSE2 is potent at high MOI and could eliminate the bacterial growth within an hour.

Cultures of *S.* Enteritidis WT (Platten) were infected with ZCSE2 at MOI 100 at 37 °C for 10 min to permit adsorption before serial dilution and incubation overnight in order to estimate the frequency of the bacteria recovered that had evaded bacteriophage infection and lysis. Under these circumstances, the survivor frequency was 0.08 ± 0.03 (*n* = 3). One hundred isolates were recovered, and 26/100 proved resistant to ZCSE2 infection, giving an estimate of the BIM frequency as 0.02.

### 3.4. Phage Genome Size Determination Using PFGE and Sequencing

To characterize ZCSE2, the genome of ZCSE2 was determined by using PFGE. The result indicated ZCSE2 was likely a DNA phage with an estimated genome size of ≈50 kb.

### 3.5. ZCSE2 Complete Genome Sequence

**Figure 2:**
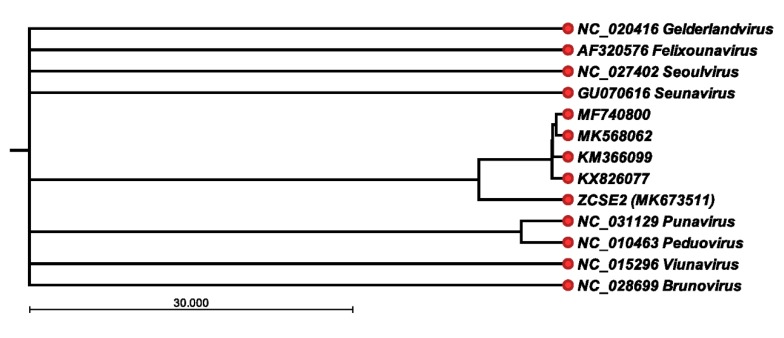
Phylogenetic relationships between *Myoviridae* infecting *Salmonella.* UPGMA (unweighted pair group method with arithmetic mean) tree based on average nucleotide identities (ANI) from pairwise comparisons of the genome sequence of ZCSE2 and bacteriophage genomes that, include reference genome sequences representing genera within the *Myoviridae* family that infect *Salmonella* (International Committee on Taxonomy of Viruses release 2018b). The bacteriophage DNA sequences are identified by their nucleotide database accession numbers.

The Illumina MiSeq platform was used to assemble a genome of 53,965 bp for phage ZCSE2. The sequence reads could be configured into a continuous circle consistent with a circularly permuted genome. However, we note the tagmentation protocol adopted for DNA sequence library preparation may not have provided the evidence required to identify genomic termini. Analysis of the nucleotide composition of ZCSE2 indicated a G+C content of 45.83%. A total of 78 putative open reading frames were identified and annotated in the sequence data (accession number MK673511). Although 33 ORFs were annotated as hypothetical proteins, it was possible to assign putative functions to 45 ORFs encoding phage structural proteins, DNA metabolic functions, and host recognition and lysis (Table 3). In common with many bacteriophages, the major capsid proteins showed sequence conservation with phages infecting taxonomically related bacteria; in this case, phages infecting *Salmonella* and other Gram-negative bacterial species showed amino acid identities between 94–39%. No genes encoding virulence factors were identified during the annotation and analysis. At the nucleotide level, four *Salmonella* phages were found to be related to ZCSE2 following BlastN analysis of the non-redundant database at NCBI: 54,894 bp UPF_BP2 (KX826077), 52,437 bp BP63 (KM366099), 50,936 bp LSE7621 (MK568062), and 52,474 bp vB_SenM_PA13076 (MF740800). Nucleotide identities of 85–87% were observed when comparing the genomes of phages UPF_BP2, BP63, LSE7621, and vB_SenM_PA13076 with coverages ranging between 69% and 74% of ZCSE2. Figure 2 presents the phylogenetic relationships of these phages referenced to genome sequences that represent genera within the *Myoviridae* family that were recorded as infecting *Salmonella* in the 2018b release of the International Committee on Taxonomy of Viruses. On the basis of average nucleotide identities from pairwise alignments, the genome sequences of ZCSE2 and sequence related phage fell within a clade that is distinct from the recognized genera of bacteriophage that replicate in *Salmonella*. However, we noted that the genome sequence of ZCSE2 showed divergence from other members of the clade.

### 3.6. ZCSE2 pH Stability

**Figure 3:**
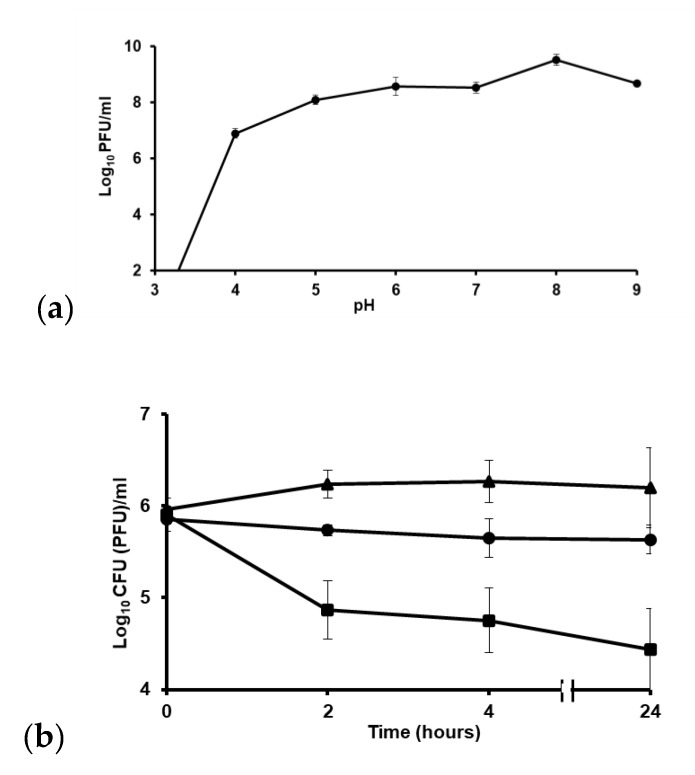
Phage stability at (**a**) different pH values shown as means ± standard error. (**b**) Phage treatment of *S.* Enteritidis WT (Platten) at 4 °C. This chart shows the drop in bacterial counts after 2 h of incubation with ZCSE2 at 4 °C. The solid circles “●” represent non-infected bacterial counts (CFU/mL), solid black squares “■” represent counts of bacteria treated with phage (CFU/mL), and the solid triangles “▲” represent the phage titer (PFU/mL). The limits of detection were 2 log_10_ CFU/mL and PFU/mL for bacteria and phage, respectively.

The ability to withstand a range of pH is one of the factors that could affect the applicability of phages. To determine the optimal pH for maximum efficacy of ZCSE2, phages were treated over a range of different pH values (2–9) and surviving titers were determined. Upon exposure to pH 3, the phage titer dropped below detection limit (3 log_10_ PFU) (Figure 3a). The maximum phage titer was recorded at pH 8 and declined at pH 9.

### 3.7. ZCSE2 Stability at Low Temperature

To test if ZCSE2 could reduce the numbers of *Salmonella* at low temperature, as would be encountered in the chilled food chain, we tested its lytic activity at 4 °C, over a 24 h period. Figure 3b shows incubation of ZCSE2 with *S.* Enteritidis WT (Platten) at 4 °C and demonstrates a reduction of 1 log_10_ CFU/mL in the bacterial count after 2 h of incubation in response to phage treatment compared to the untreated control (*p* = 0.01). The phage treated culture remained >1 log_10_ CFU/mL lower than the control over 24 h. The phage titer remained stable at 4 °C.

### 3.8. ZCSE2 Characterization and Imaging Bacterial Lysis with TEM

**Figure 4:**
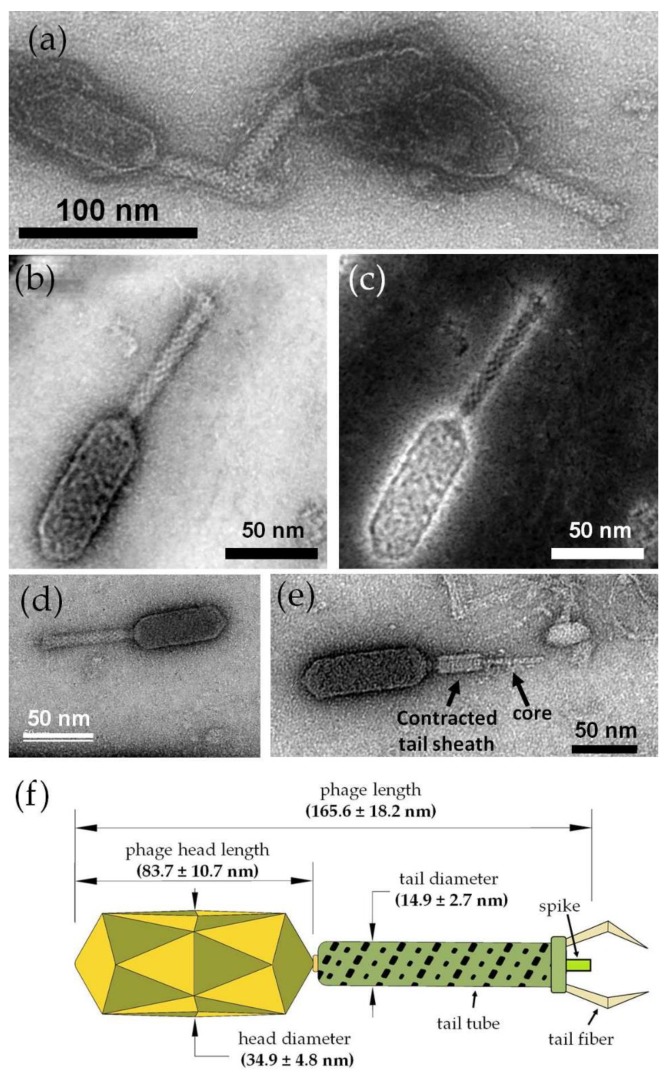
TEM micrographs and investigations of the ZCSE2 bacteriophage. (**a**) Image showing a group of three negatively stained phages acquired by parallel illumination in TEM mode using the Philips CM12 microscope. (**b**) Bright-field scanning transmission electron microscopy (BF-STEM) image acquired by the probe-corrected Titan microscope for an isolated ZCSE2 bacteriophage showing the main morphological components: long head, collar, tail tube, tail fibers, baseplate, and a spike. (**c**) STEM-high annular angle dark field (HAADF) image acquired simultaneously with the BF-STEM image in STEM mode with the double-correction of the spherical aberration in both probe and imaging plans. (**d**) High-resolution TEM image of the same phage acquired with single-correction in the imaging plan only in TEM mode using a US1000FTXP CCD camera. (**e**) TEM image showing a fully contracted tail sheath with the core visible, where the contracted tail diameter (13.1 nm) was less than the average (14.9 ± 2.7 nm) of the uncontracted tail diameter, as expected, due to the squeezing of the helical tail tube. (**f**) Illustration figure showing the main dimensions of the ZCSE2 bacteriophage, as measured from the TEM images acquired during screening the sample.

Parallel illumination TEM mode revealed the existence of negatively stained groups of the ZCSE2 phages (Figure 4a), from which a well-separated bacteriophage could be imaged at higher magnification (Figure 4b) using a double-corrected microscope in STEM mode. The STEM image revealed that ZCSE2 has a complex structure that corresponds with the dsDNA type (T-even phages) of the *Myoviridae* family with a characteristic elongated head, collar, tail tube, tail fibers, baseplate, and a spike. The same phage was also imaged using a STEM-HAADF detector and image-corrected electron beam in TEM mode in Figure 4c,d, respectively. It was observed that the STEM mode provided better resolution and contrast for the same feature due to the benefit of correction the spherical aberrations of the electron probe in both the probe forming and imaging planes.

The contractile nature of the ZCSE2 tail is presented in Figure 4e, which shows a contracted tail sheath relative to the central tail tube. The contracted tail diameter was observed to have a lower diameter (13.1 nm) than the average values of the non-contracted tails (14.9 ± 2.7 nm) because of the expected contraction mechanism that combines both linear and rotational motions of the outer tail tube. Figure 4f illustrates the main dimensions of the ZCSE2 as measured from TEM images (see Figure S1 and Table S2). It shows that the phage had an approximate 1:1 ratio of the head length to the tail length, whereas the head diameter was a little more than twice the tail diameter, with a ratio of 2.3:1. In this study, the phage length was measured from the head to the spike without including the length of the fibers because these may be truncated in the image.

**Figure 5:**
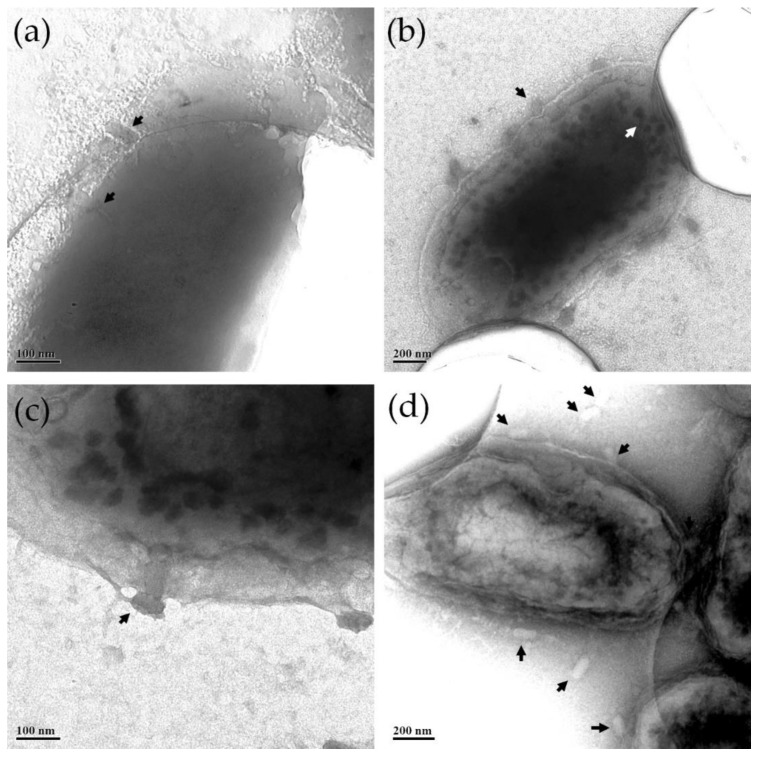
TEM images of different bacteria cells infected by the ZCSE2 phages. The images were contrasted to show (**a**) two ZCSE2 phages (black arrows) attached to the bacteria wall; (**b**) infected bacteria, but not yet lysed, with empty capsids (black arrow) attached to its wall, with the particles with dark contrast inside the bacteria (white arrow) representing nascent phages being formed in an earlier stage inside the bacteria; (**c**) a close up image showing an empty capsid attached to the bacteria wall and nascent phages inside; (**d**) a few mature phages (black arrows) surround a lysed bacteria with crumpled walls.

ZCSE2-infected *S.* Enteritidis WT (Platten) cells were imaged using the transmission electron microscope in parallel illumination. At an early stage of infection process, phages became attached to the bacterial cell wall (Figure 5a) before the phages injected their DNA into the cell, and empty capsids could be observed as being attached to the cell wall (black arrows in Figure 5b,c). Post-replication nascent phages were formed inside the infected cell (Figure 5b, white arrow) until rapture of the bacterial cell wall released multiple phages from the lysed bacteria (Figure 5d).

### 3.9. ZCSE2 Characterization and Imaging Bacterial Lysis with AFM

Atomic force microscopy imaging was carried out on a HOPG hydrophobic surface that maintains the water content to prevent damage over the course of the experiment [14,15]. Typically, the AFM examination was performed over 1–2 h incubation at 20 to 25 °C after dispensing bacteria and phages on the HOPG surface. Figure S2 illustrates how the AFM microscope scans the ZCSE2 bacteriophage particle on an atomically flat highly oriented pyrolytic graphite (HOPG) surface. The AFM scanning profile reflects only the correct measurement of the phage head diameter relative to the flat surface. AFM scanning confirmed the morphology revealed by the TEM, and a phage head diameter of 46.4 nm was determined using the cross-section perpendicular to the phage head (Figure S3). AFM imaging of the host bacteria *S.* Enteritidis WT (Platten) revealed the dimensions of the bacillus (0.2 µm wide and 1.2 µm long) in the absence of infection (Figure S4).

**Figure 6:**
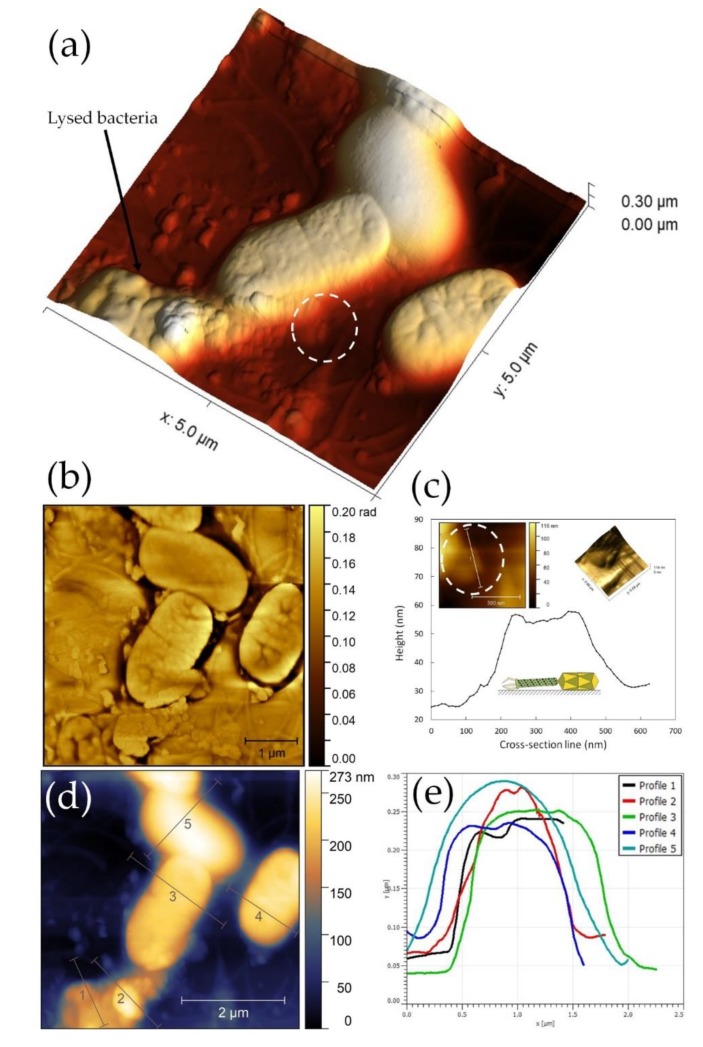
Atomic force microscopy (AFM) analysis of bacterial lysis. (**a**) A 3D image of four cells on highly oriented pyrolytic graphite (HOPG) surface. The bacteria on the bottom-left was found to be ruptured and numbers of bacteriophages came out from it and attached to the neighbor bacteria. (**b**) AFM phase image of ruptured bacteria showed a difference in contrast between the raptured bacterial cell wall and the phages that emerge from the torn region. The difference in color of the phase image can be interpreted as a differences in the sample stiffness of bacteria and phages, which produced a phase shift of the AFM probe vibration during scanning. (**c**) Cross-sectional profile of the bacteriophage indicated in the circle in panel (**a**), where the left and right inserts are respectively the 2D and 3D AFM images of the bacteriophage. (**d**) 2D AFM image of the same site with the corresponding cross section lines. (**e**) Profiles of the cross-section lines in panel (**d**) show the height gradient related to the infection stage. Profile 1 is a lysed cell, profile 2 represents accumulated phages from the lysed cell, profiles 3 and 4 are infected cells, and profile 5 represents an uninfected cell. The raptured cell in the bottom-left section—profile 1—has the lowest height. It is also notable that the cells in the late infection stage have surface roughness and wrinkles.

AFM examination of the ZCSE2 lytic cycle after incubation with *S.* Enteritidis WT (Platten) was undertaken using a group of four bacteria identified in an optical survey of the HOPG surface (see Figure S5). The 3D image presented in Figure 6a shows cells in the process of infection and phage-mediated lysis. Figure 6b shows an AFM phase image collected simultaneously, where the color contrast in the AFM phase differentiates the lysis event at the surface of the bacterium [14]. Lysis is associated with the emergence of ZCSE2 phage, as indicated in the circle in Figure 6a. The maximum AFM height of this phage was found to be 57 nm, as shown in the corresponding location of Figure 6c, which is consistent with the phage dimensions measured by AFM and TEM.

The height of the ruptured bacterium (profile 1, Figure 6e) measured in the cross-section was lower than the other three cells as a result of phage release after lysis, which accumulated to form the height measured in profile 2. The change in height was accompanied by a rougher surface compared to non-infected bacteria in the 2D and 3D and phase images (Figure 6b,d). The two cells in the center of the image also exhibited profiles that were rougher (profiles 3 and 4 in Figure 6d) than the cell at the top of the image (profile 5 in Figure 6d). The central two cells were infected and the surface roughness observed a consequence of late infection pre-cell rupture.

## 4. Discussion

Developing alternative non-antibiotic biocontrol techniques for eliminating pathogenic microorganisms from clinical settings, agricultural production environments, and retail food products constitutes an important avenue of research in antimicrobial development [31]. Phages that conform to the criteria of being strictly lytic (virulent), have a broad target host-range, and display a high degree of efficacy against their microbial hosts are ideal candidates for phage therapy and biosanitization applications. Here, we report the structural and functional characteristics of a novel broad host-range *Salmonella*-specific bacteriophage ZCSE2. A combination of TEM and AFM were employed to determine morphological features of ZCSE2 and visualize the infection process using *S.* Enteritidis as the host strain. The genome sequence of ZCSE2 has few homologues in the database, but collectively the genomes of these bacteriophage are distinct from other genera of the *Myoviridae* and have the potential to form the basis for a new taxonomic subclass family (Figure 2). In planktonic growth, ZCSE2 was observed as reducing the target *S.* Enteritidis population to below the limits of detection (10^2^ CFU/mL) when applied at MOIs greater than 1.

Characterization of the ZCSE2 phage by TEM revealed the phage morphology and dimensions. Sub-nanometer spatial resolution (≈2.1 Å) was achieved using the probe-corrected STEM beam, which was higher than the uncorrected TEM in parallel illumination mode. The STEM images of ZCSE2 phage indicated that the phage was a dsDNA type from the *Myoviridae* family featuring a long crystal head and a contractile tail with a spike and tail fibers. The central tail fiber was observed when the outer tail tube fiber was contracted relative to its head. Structural similarities to the *Myoviridae*, notwithstanding the genome structure and sequence, indicated the fact that ZCSE2 is a member of a small group of novel phages, the structures and lifecycle of which have hitherto not been investigated. Snapshots of the lysis process were captured in TEM images due to the variations in the stage of infection. TEM images showed the capsids attached to the cell wall of the host bacteria and nascent phage production inside the cell. These observations were consistent with a virulent lifestyle and the suitability of the phage for biocontrol applications.

An important advantage of the AFM technique is that it can efficiently examine biological samples in atmospheric pressure [17]. This gives privileges to the AFM technique to maintain hydration of biological samples in contrast to electron microscopy techniques that require vacuum and sample preparation using chemicals for fixing and staining the phages on the TEM grids. Despite the high vertical resolution of the AFM (0.2 nm), the measurements of the phage dimensions are only reliable for the phage head diameter. The phage head diameter measured by the vertical AFM resolution was within the distribution measured by TEM microscopy (Figure S1e). However, AFM measurement of the phage length can have artifacts due to the contractile nature of the ZCSE2 phage tail, in addition to the effect by the AFM probe radius on the lateral resolution direction. The probe radius adds extra length, which results in an overestimate along the phage axis, as shown in the model presented in Figure S2 and experimentally observed in Figure S3. The phage tail diameter is located within the range of vertical resolution but cannot be accurately measured because the tail orientation is not horizontally parallel to the HOPG flat surface. However, using the AFM, we were able to examine the lytic cycle and the effects on bacterial host dimensions. Infected bacteria were found to have less AFM height and a rough cell surface compared to a non-infected cell. Bacterial lysis was characterized by a decline of the cell height and the observation of phage release (the size suggests tens of virions) to propagate the infection of surrounding bacteria.

The ZCSE2 bacteriophage displayed high efficacy in reducing the counts of viable bacteria in vitro over a wide host range that included various *Salmonella* serovars. With the application of high concentrations of bacteriophages (MOI of 10), ZCSE2 was demonstrated to reduce the numbers of *S.* Enteriditis by ≥5.5 log_10_ CFU/mL. This reduction in viability was recorded over 40 min, which likely relates to the loss of host viability by a combination of infection and non-infectious killing by an established phenomenon called “lysis from without”, where many phages become absorbed to bacterial cells causing lysis without the release of new phages [32]. The use of high phage concentrations increases the chance of phage attaching to their host cells and, accordingly, significantly increases their effectiveness in reducing the numbers of target host over a short time period [9,33]. This is important because the use of single high dose treatment does not result in the generation of phage-resistant bacterial mutants, which is considered a limitation of phage therapy. We estimated the BIM frequency as ≈2%, which compares favorably with other bacteriophage therapy studies [34]. Bacteriophage ZCSE2 remained stable at a low temperature (4 °C) and was able to bring about a reduction in viable *S.* Enteritidis compared to the control. These results support previous reports of low temperature phage lysis of host bacteria [12]. Significant reductions in *Salmonella* counts are encouraging for their adoption for commercial use in the control of bacterial contamination in food products, as concluded by several studies [8,12,33], especially with foods that are considered sources of salmonellosis, such as poultry, eggs, and milk [35,36].

Environmental stability is considered a limiting factor that defines the ability of phage to sustain efficacy over periods of transport and storage before use in decontamination. Bacteriophage ZCSE2 was robust in this context, with prominent stability over a range of pH conditions. Collectively, ZCSE2 was an efficient bacteriophage with high stability, strong lytic ability, and a broad host range enough to qualify it for application in the decontamination of *S. enterica* serovars of significance to human and animal health.

## Figures and Tables

**Table 1:** Antibiotic susceptibility profile of *Salmonella* Enteritidis WT (Platten).

Class	Antibiotics	Diameter of Inhibition Zone (cm)	Results
Cephalosporins Second generation	Cefoxitin	2.2	Intermediate
	Cefaclor	0.8	Resistant
Cephalosporins Third generation	Cefotaxime	2.2	Resistant
	Chloramphenicol	2.9	Susceptible
	Ceftazidime	2.3	Susceptible
	Ceftriaxone	3.1	Susceptible
Macrolides	Clarithromycin	0.9	Resistant
	Erythromycin	0.8	Resistant
	Vancomycin	0	Resistant
	Azithromycin	1.4	Intermediate
Quinolones	Levofloxacin	3.4	Susceptible
	Ciprofloxacin	3.0	Intermediate
Penicillins	Piperacillin	2.5	Susceptible
	Oxacillin	0	Resistant
Oxazolidinones	Linezolid	0.8	Resistant
Lincosamides	Clindamycin	0	Resistant
Nitrofurans	Nitrofurantoin	1.3	Resistant
Carbapenems	Ertapenem	3.0	Susceptible
Aminoglycosides	Amikacin	1.9	Susceptible
Aminocoumarin	Novobiocin	1.2	Resistant
Tetracyclines	Tetracycline	2.8	Susceptible

**Table 2:** Lysis range of *Salmonella* phage isolates.

*Salmonella* Strains	ZCSE2	ZCSE3	ZCSE4	ZCSE5
*S.* Agama WT	+	+	+	−
*S.* Togla Amersham 5.8.95	+	−	−	+
*S.* Amsterdam WT	+	−	−	−
*S.* Typhimurium DT104 NCTC 13348	+	+	−	−
*S.* Atlanta NCTC 9986	+	+	+	−
*S.* Typhimurium LT2	+	+	−	−
*S.* Bareilly NCTC 5745	+	+	−	−
*S.* Typhimurium WT Rawlings	+	+	−	−
*S.* Derby WT	+	+	−	−
*S.* Typhimurium S21344	+	+	−	−
*S.* Enteritidis HOOO WT	+	+	−	−
*S.* Typhimurium WT Turner	+	+	−	−
*S.* Enteritidis WT (Platten)	+	+	−	−
*S.* Typhimurium U288	+	+	−	−
*S.* Enteritidis SA029	+	+	−	−
*S*. Virchow WT	+	+	+	−
*S.* Enteritidis WT Harrison	+	+	−	−
*S.* Hadar WT	+	+	+	−
*S.* Infantis NCTC 6903	+	−	−	+
*S.* Kedougou	+	−	−	+
*S*. Kubacha WT	+	+	−	−
*S*. Montevideo NCTC 5797	+	−	−	−
*S*. Montevideo WT	+	−	−	−
*S*. Senftenburg WT	−	−	−	−
*S.* Thompson NCTC 2252	+	+	−	−

+ indicates plaque formation, − indicates no plaque formation.

**Table 3:** Phage ZCSE2 proteins with putative functions.

Protein ID	Chromosomal Loci (nt)	Putative Function
QBZ70504	1–978	Major capsid protein
QBZ70505	1051–1425	HNH endonuclease
QBZ70506	1455–1955	DCTP pyrophosphatase
QBZ70507	1955–2455	DNA primase
QBZ70508	2448–2825	Head-to-tail interface protein
QBZ70510	3276–3896	Minor tail fiber
QBZ70511	3911–6853	Tail protein
QBZ70512	6923–7564	Head-to-tail interface protein
QBZ70513	7577–9451	Sheath structural protein
QBZ70514	9539–10,678	Cell adhesion protein
QBZ70515	10,689–11,120	DNA helicase
QBZ70516	11,137–11,565	Membrane-associated initiation of head vertex
QBZ70517	11,616–11,750	RecA-like recombination protein
QBZ70518	11,731–13,392	DNA polymerase
QBZ70520	14,619–15,569	RegA-like translational repressor
QBZ70521	15,559–16,203	Baseplate assembly V
QBZ70522	16,212–16,583	Clamp-loader subunit
QBZ70523	16,587–17,750	Sliding clamp DNA polymerase
QBZ70524	17,743–18,396	RNA polymerase binding protein
QBZ70525	18,389–19,738	Tail fiber
QBZ70526	19,738–20,280	Tail fiber assembly protein
QBZ70527	20,283–20,792	Tail fiber chaperone
QBZ70528	20,894–21,157	dsDNA binding protein
QBZ70529	21,201–21,677	Lysozyme
QBZ70530	21,656–21,985	DUF2570 domain-containing protein
QBZ70531	22,203–22,427c	TraR/DksA family transcriptional regulator
QBZ70533	22,711–24,681c	DNA polymerase 1
QBZ70534	24,678–25,676c	DNA polymerase beta subunit
QBZ70535	25,709–26,593c	Thymidylate synthase
QBZ70536	26,593–27,186	NTP pyrophosphohydrolase
QBZ70541	28,955–30,628c	ATP-RNA helicase
QBZ70543	30,835–31,710c	Cas4 family exonuclease
QBZ70544	31,712–32,176c	Deoxycytidylate deaminase
QBZ70545	32,237–32,776c	DUF669 domain-containing protein
QBZ70546	32,881–33,771c	σ factor for late transcription
QBZ70550	35,919–36,215c	Glyoxalase
QBZ70552	36,284–36,616c	Host-nuclease inhibitor protein
QBZ70558	38,983–39,315	Glutaredoxin
QBZ70559	39,290–41,824	Anaerobic NTP reductase small subunit
QBZ70560	42,319–42,747	Protector of prophage-induced early lysis
QBZ70574	47,327–47,998	Dihydrofolate reductase
QBZ70575	47,995–48,495	Thymidylate kinase
QBZ70579	50,055–51,503	Terminase large subunit
QBZ70580	51,505–53,067	Portal protein
QBZ70581	53,246–53962	Scaffold protein

Nucleotide locations with suffix c indicate the reading frame is located on the complementary strand.

## Supplementary Materials

Supplementary materials can be found at https://www.mdpi.com/1999-4915/12/4/424/s1.
