# Supplementary material for: Bacteriophage ZCSE2 is a Potent Antimicrobial against Salmonella enterica Serovars: Ultrastructure, Genomics and Efficacy
Source: Viruses. 2020 Apr 9;12(4):424. doi: 10.3390/v12040424 (PMC7232530; doi:10.3390/v12040424)
Supplement: Supplementary file 1 [file viruses-12-00424-s001.pdf]

Supplementary Figures

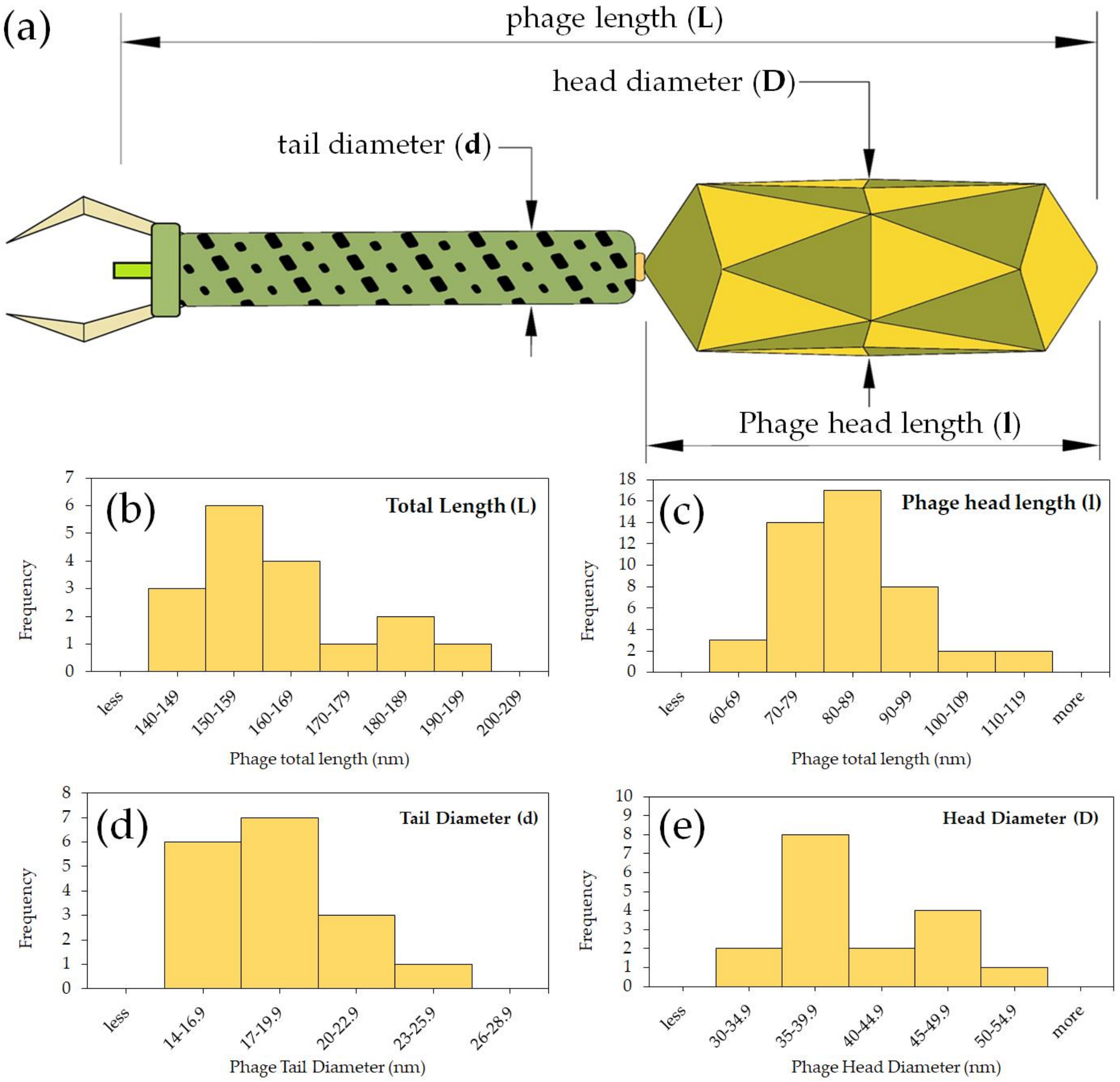

Figure S1. Phage dimensions distribution as measured during screening the sample by TEM in parallel illumination; (a) illustration figure shows the main dimension symbols and how they are measured; histogram of the (b) total phage length; (c) phage head length; (d) phage tail diameter; (e) phage head diameter.

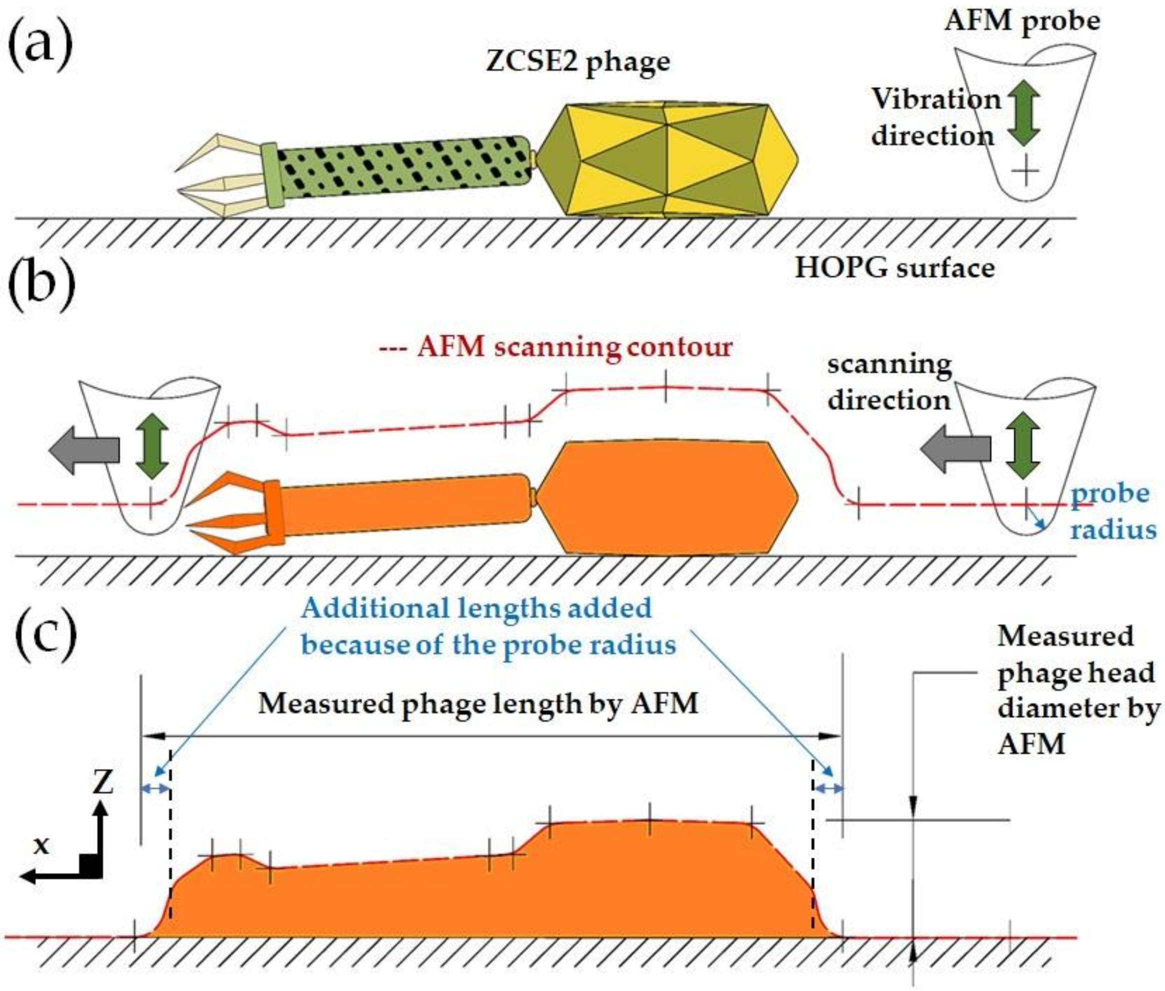

Figure S2. Illustration figure shows how the AFM scans a ZCSE2 bacteriophage placed on an atomically flat surface; First, (a) the ZCSE phage is drop casted on flat HOPG surface and the AFM probe is prepared to do the scan; (b) Then the AFM tip is used to trace the outer contour of the bacteriophage in the direction of scanning with a non-contact mode; (c) The vibration changes of the AFM cantilever is traced and processed to provide the outer contour of the phage profile; The AFM contour gives an accurate measurement of the phage head diameter because it sets flat relative to the HOPG flat surface; The phage length is usually measured larger than the real value because it is affected experimentally by the probe geometry.

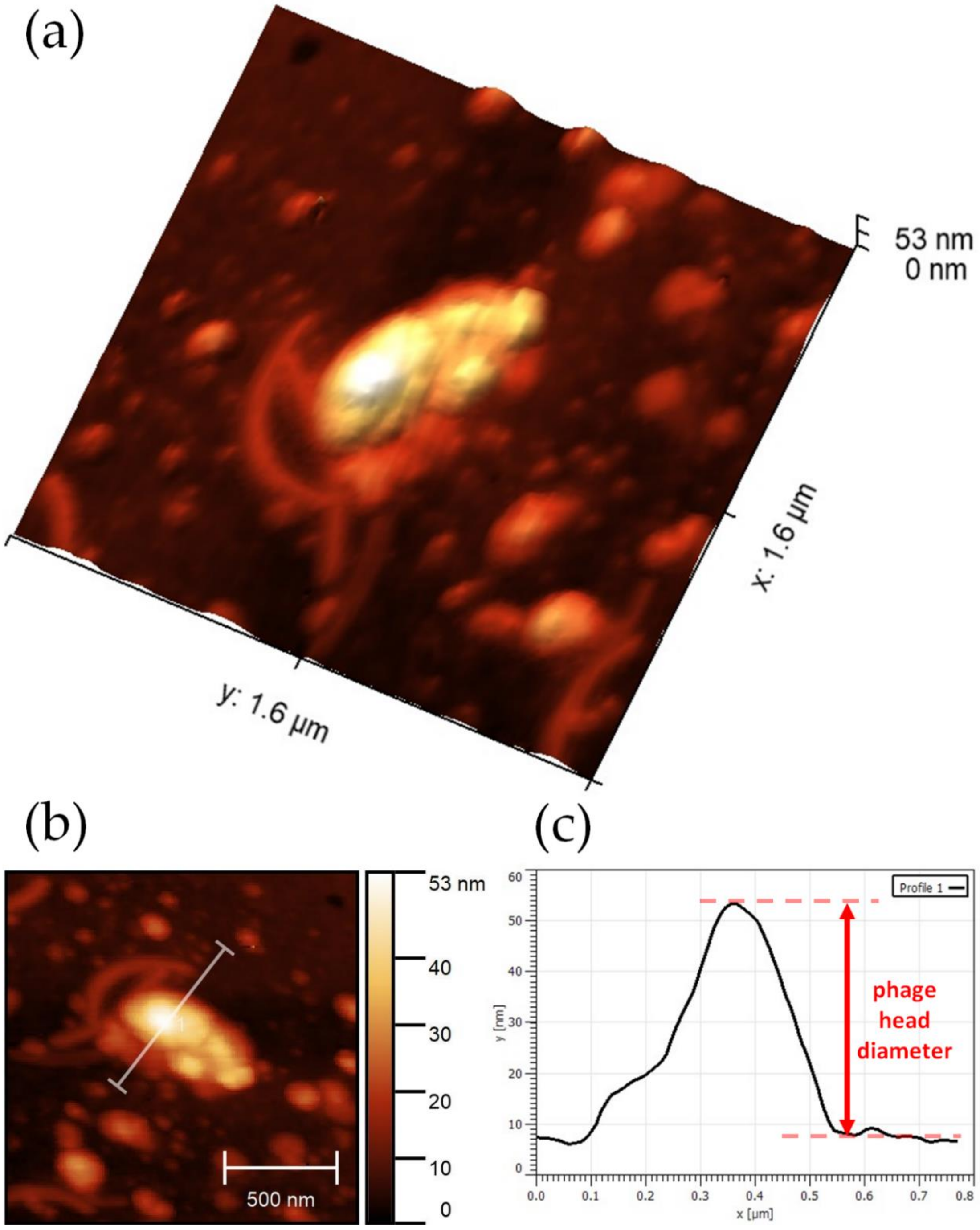

Figure S3. AFM of a ZCSE2 bacteriophage placed on HOPG surface. (a) 3D topography surface of the shows that the ZCSE2 has long head, tail, and a baseplate; (b) 2D view shows a section line where the profile in (c) is collected; The phage head diameter is found to be ~ 46.4 nm that fits within the TEM measurements.

(a)

(b)

(c)

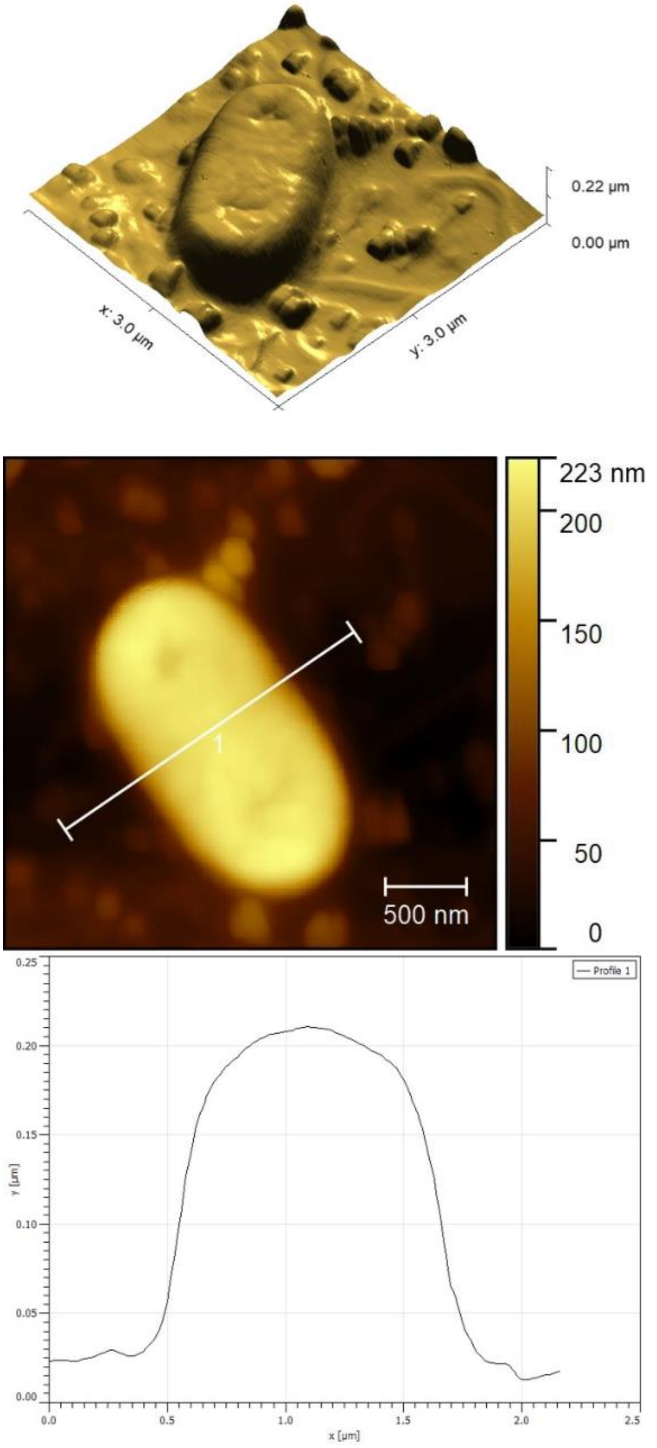

Figure S4. AFM imaging of Salmonella Enteritidis WT Bacteria on the on HOPG surface with average height and width of 0.2 µm and 1.2 µm length, (a) 3D view, (b) 2D view with section line 1 across the bacteria, (c) profile of the cross-section line 1 in the 2D image.

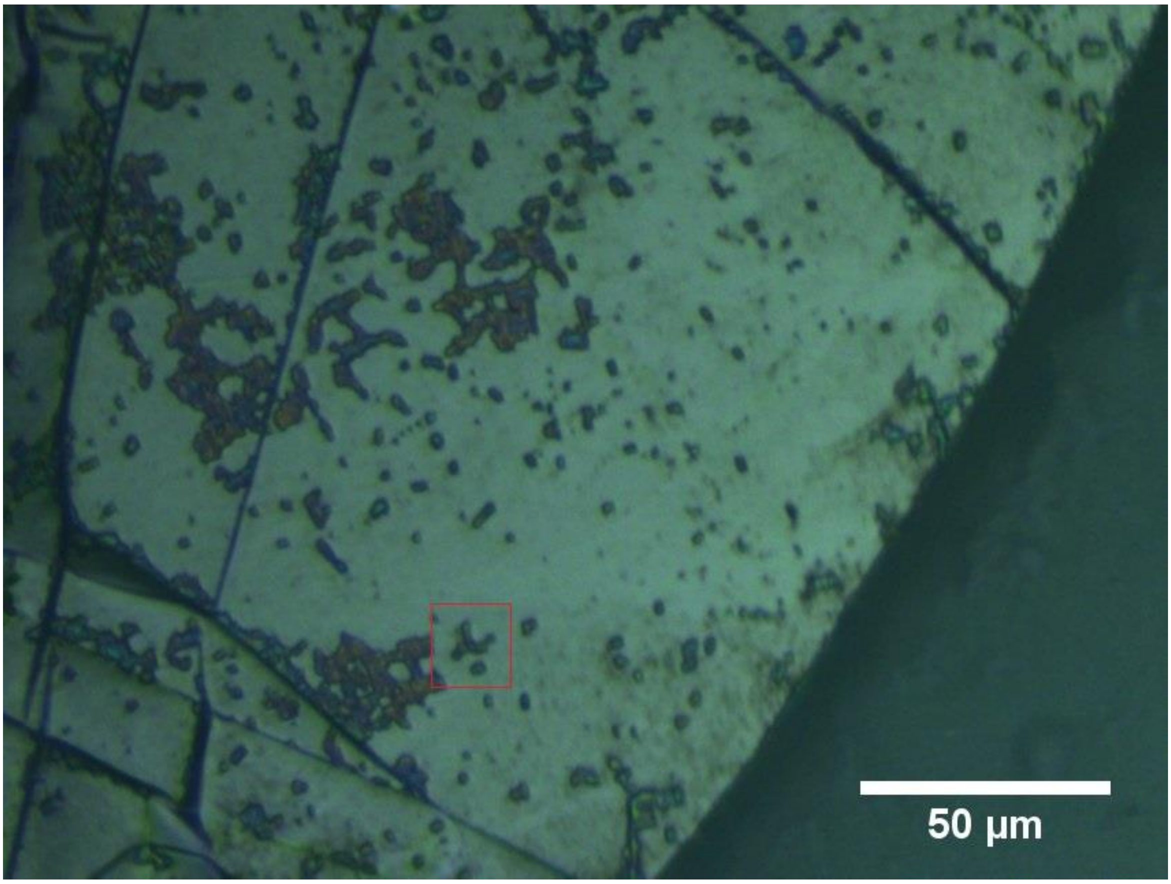

Figure S5. Optical microscope image of the bacteria with phages dispersed on HOPG surface. The red box is the position where the AFM images are collected in Fig S-9.

Supplementary Tables

Table S1. The EOP for ZCSE2 phage against a variety *Salmonella enterica* strains

Salmonella **Isolate**	**Average PFU/ml (±SD)**	**EOP**
Salmonella Agama WT	5.2 (± 0.32)	0.54
Salmonella Amsterdam WT	5.3 (± 0.25)	0.54
Salmonella Atlanta NCTC 9986	7.2 (± 0.17)	0.74
Salmonella Bareilly NCTC 5745	8.1 (± 0.61)	0.84
Salmonella Derby WT	7.4 (± 1.21)	0.77
Salmonella Enteritidis HOOO WT	5.3 (± 0.3)	0.55
Salmonella Enteritidis SA029	6.9 (± 0.4)	0.72
Salmonella Enteritidis Harrison WT	7.8 (± 0.91)	0.81
Salmonella Hader WT	9.6 (± 0.65)	0.10
Salmonella Infantis NCTC 6903	7.1 (± 0.61)	0.73
Salmonella Kubacha WT	8.2 (± 1.04)	0.85
Salmonella Montevideo NCTC 5797	9.6 (± 0.75)	0.99
Salmonella Montevideo WT	6.5 (± 0.5)	0.68
Salmonella Thompson NCTC 2252	8.5 (± 0.25)	0.88
Salmonella Togla Amersham 5.8.95	5.4 (± 0.4)	0.55
Salmonella Typhimurium DT04 NCTC 13348	8.5 (± 0.25)	0.88
Salmonella Typhimurium LT2	7 (± 0.35)	0.72
Salmonella Typhimurium Rawlings WT	6.9 (± 0.67)	0.72
Salmonella Typhimurium S21344	4.4 (± 0.23)	0.46
Salmonella Typhimurium Turner WT	3.1 (± 0.17)	0.32
Salmonella Typhimurium U288	6.0 (± 0)	0.62
Salmonella Virchow WT	4.8 (± 0.26)	0.50

Table S2. Statistical analysis of phage dimensions measured from TEM

**Dimention**	**Number of counts**	**Mean (nm)**	**Standard deviation (nm)**
Length (L)	17	165.6	18.2
Head Diameter (D)	17	34.9	4.8
Tail Diameter (d)	17	14.9	2.7
Head length	46	83.7	10.7
